# Multi-level engineering facilitates the production of phenylpropanoid compounds in tomato

**DOI:** 10.1038/ncomms9635

**Published:** 2015-10-26

**Authors:** Yang Zhang, Eugenio Butelli, Saleh Alseekh, Takayuki Tohge, Ghanasyam Rallapalli, Jie Luo, Prashant G. Kawar, Lionel Hill, Angelo Santino, Alisdair R. Fernie, Cathie Martin

**Affiliations:** 1 Department of Metabolic Biology, John Innes Centre, Norwich Research Park, Norwich NR4 7UH, UK; 2 Max-Planck-Institute of Molecular Plant Physiology, Am Muehlenberg 1, 14476 Potsdam-Golm, Germany; 3 The Sainsbury Laboratory, Norwich Research Park, Colney, Norwich NR4 7UH, UK; 4 National Key Laboratory of Crop Genetic Improvement and National Center of Plant Gene Research (Wuhan), Huazhong Agricultural University, 430070 Wuhan, China; 5 Division of Crop Improvement, Indian Council of Agricultural Research – Central Potato Research Institute, Shimla 171001, Himachal Pradesh, India; 6 Institute of Sciences of Food Production C.N.R. Unit of Lecce, Via Monteroni, 73100 Lecce, Italy

## Abstract

Phenylpropanoids comprise an important class of plant secondary metabolites. A number of transcription factors have been used to upregulate-specific branches of phenylpropanoid metabolism, but by far the most effective has been the fruit-specific expression of *AtMYB12* in tomato, which resulted in as much as 10% of fruit dry weight accumulating as flavonols and hydroxycinnamates. We show that AtMYB12 not only increases the demand of flavonoid biosynthesis but also increases the supply of carbon from primary metabolism, energy and reducing power, which may fuel the shikimate and phenylalanine biosynthetic pathways to supply more aromatic amino acids for secondary metabolism. AtMYB12 directly binds promoters of genes encoding enzymes of primary metabolism. The enhanced supply of precursors, energy and reducing power achieved by AtMYB12 expression can be harnessed to engineer high levels of novel phenylpropanoids in tomato fruit, offering an effective production system for bioactives and other high value ingredients.

## Abstract (web summary)

Metabolic engineering offers an effective strategy for producing valuable bioactive compounds in plants. Here, the authors show that by harnessing transcriptional regulation of carbon flux, tomato fruit metabolism can be optimized for the production of phenylpropanoids.

## Graphical abstract

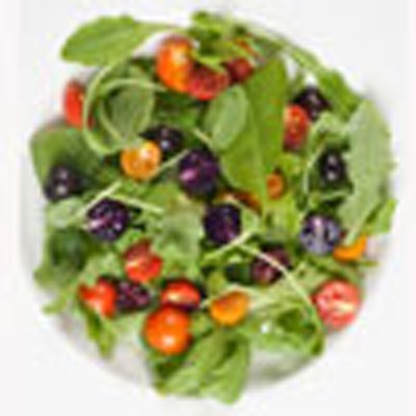

The use of transcription factors (TFs) to activate or suppress secondary metabolism has provided effective strategies to engineer plants enriched in valuable secondary metabolites^1^,^2^. However, the importance of primary metabolism to engineer high levels of plant secondary metabolites has been appreciated only more recently. Expression of AtMYB12, a TF regulating flavonol biosynthesis in *Arabidopsis thaliana*, under the control of the fruit-specific E8 promoter, activated the expression of genes encoding enzymes of flavonol and hydroxycinnamic ester biosynthesis in tomato resulting in accumulation of exceptionally high levels of both flavonols and caffeoyl qunic acids, amounting to as much as 100 mg g^−1^ dry weight (DW)^3^. This suggested that AtMYB12 might be a useful tool for engineering phenylpropanoid metabolism.

Flavonoids along with other phenylpropanoids are synthesized from phenylalanine. Erythose-4-phosphate, produced from the oxidative pentose phosphate pathway (OPPP) and phosphoenolpyruate from glycolysis, are the precursors for the seven-step, shikimate pathway^4^ which supplies phenylalanine for phenylpropanoid metabolism. The enzyme 3-deoxy-D-arabino-heptulosonate 7-phosphate synthase (DAHPS) is a key determinant of the flow of carbon into the shikimate pathway^5^,^6^ and phenylalanine along with other aromatic amino acids are the products of this pathway^4^. General phenylpropanoid metabolism begins with phenylalanine, and involves the activity of three enzymes (phenylalanine ammonia lyase (PAL), cinnamate 4-hydroxylase (C4H) and 4-coumaroyl CoA ligase (4CL)) to generate *p-*coumaroyl CoA, the activated intermediate for the various branches of phenylpropanoid metabolism^7^ (Fig. 1).

We investigated how AtMYB12 induces phenylpropanoid biosynthesis so effectively in tomato fruit. Our data indicate that AtMYB12 can induce both primary and secondary metabolism and binds directly to the promoters of genes encoding enzymes of primary metabolism, including DAHPS and the gene encoding plastidial enolase (ENO). Through its ability to regulate metabolism at multiple levels, AtMYB12 can be used as a general tool to engineer appreciable levels of novel phenylpropanoids in tomato.

## Results

### AtMYB12 activates genes in primary and secondary metabolism

**Figure 2:**
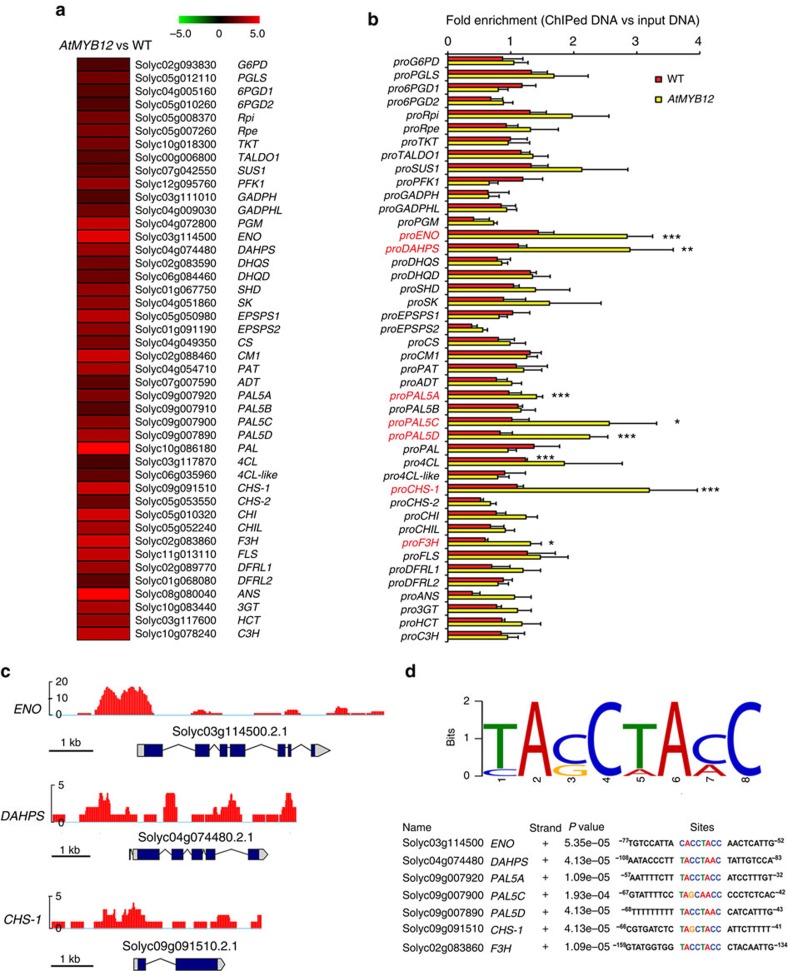
AtMYB12 binds directly to the promoter regions of genes encoding enzymes of both primary and secondary metabolism to promote flavonoid biosynthesis in tomato. (**a**) RNA-seq showed that genes involved in the pentose phosphate pathway, glycolysis, the shikimate pathway and flavonoid biosynthesis were upregulated in *AtMYB12* tomatoes compared with controls. The heat map compares transcript levels in *AtMYB12* tomatoes with those in WT fruit. Absolute values are scaled by log2. Gene IDs and abbreviations are explained in Supplementary Table 6. (**b**) AtMYB12 binds directly to the promoter regions of seven genes encoding enzymes of both primary and secondary metabolism (highlighted in red), as shown by ChIP-qPCR analysis of AtMYB12 binding in the promoter regions of genes shown in **a**. *ACTIN* was used as internal control. The promoter sequence enrichment compared with *ACTIN* was calculated for each sample. Data are presented as the ratio between ChIPed DNA and Input DNA. Error bars represent s.e.m. (*n*=3). *(*P*<0.15), **(*P*<0.10) and ***(*P*<0.05) (Student's *t*-test) indicate significant enrichment compared with input DNA. (**c**) ChIP-sequencing data of AtMYB12 binding sites in the *ENO*, *DAHPS* and *CHS1* promoters. Scale bars, 1 kb. (**d**) Predicted binding motif of AtMYB12 in tomato. Sequence alignment was performed using MEME. Numbers indicate the nucleotide positions from the start of transcription.

To understand better the activity of AtMYB12 in regulating flavonoid metabolism, we undertook RNA-seq on both ripe wild type (WT) and *AtMYB12* tomatoes. Expression of *AtMYB12* in tomato increased transcript levels of genes involved in primary metabolism, in addition to genes encoding the enzymes of flavonol biosynthesis. Compared with WT fruit, nearly all the genes encoding enzymes of glycolysis, the pentose phosphate pathway and the shikimate pathway were expressed more highly in *AtMYB12* tomato fruit than in control fruit (Fig. 2a and Supplementary data).

### AtMYB12 binds to the promoters of the genes it activates

We investigated which of the promoter regions of those genes upregulated in *AtMYB12* tomato were directly bound by the AtMYB12 TF by chromatin immunoprecipitation (ChIP) using an antibody designed to detect the C-terminus of AtMYB12 (Methods). ChIP-quantitative PCR (qPCR) data showed that the promoters of five genes in secondary metabolism, *PAL5A*, *PAL5C*, *PAL5D*, *CHS1* and *F3H*, were significantly enriched in chromatin immunoprecipitated using the MYB12 antibody in *AtMYB12* tomatoes compared with the input DNA. In addition to these known targets of AtMYB12, two genes encoding enzymes of primary metabolism, enolase (*ENO*) and DAHPS, also showed significant enrichment of their promoter regions in chromatin immunoprecipitated from *AtMYB12* fruit (Fig. 2b). This indicated direct binding of AtMYB12 to promoters of the genes encoding plastidial ENO and DAHPS as well as PAL5 (A, C, D), CHS, and F3H. ChIP-seq confirmed enrichment of the promoters of these genes encoding enzymes of primary metabolism in the chromatin immunoprecipitated by the MYB12 antibody (Fig. 2c).

**Figure 3:**
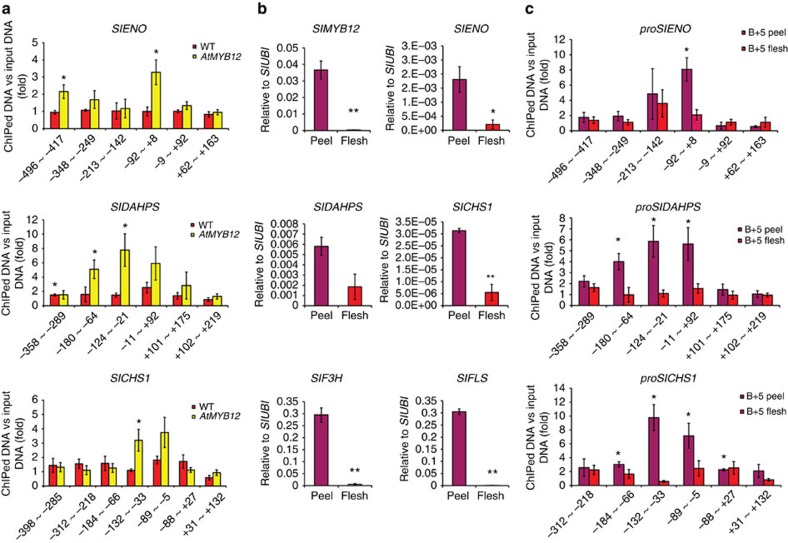
AtMYB12 and SlMYB12 bind directly to the promoter regions of *SlENO*, *SlDAHPS* and *SlCHS1*. (**a**) Binding of AtMYB12 to the regions of *SlENO*, *SlDAHPS* and *SlCHS1* was analysed by ChIP-qPCR. Pericarp (peel plus flesh) samples of WT and *AtMYB12* fruit were harvested at 3 days after breaker. The numbers on the horizontal axis below the bars correspond to the left and right border of the amplified region relative to the transcription start site and bars indicate means and s.e.m.'s (*n*=3); asterisks indicate significant differences compared with the negative control (*P* value<0.05, Student's *t*-test). (**b**) S*lMYB12* expression is predominantly in the peel of WT tomato fruit and is associated with elevated expression of genes involved in both primary and secondary metabolism. Expression of *SlMYB12*, *SlENO*, *SlDAHPS*, *SlCHS1*, *SlF3H* and *SlFLS* was measured by qRT-PCR in both peel and flesh of WT fruit at 5 days after breaker. Error bars show s.e.m. (*n*=3), *(*P*<0.05) and **(*P*<0.01) indicate significant differences (Student's *t*-test). (**c**) Binding of SlMYB12 to the promoters of *SlENO*, *SlDAHPS* and *SlCHS1* was confirmed by ChIP-qPCR. Peel and flesh samples of WT fruit were harvested at 5 days after breaker. The numbers on the horizontal axis below the bars correspond to the left and right borders of the amplified regions relative to the initial transcription start site, and bars indicate means and s.e.m.'s (*n*=3); asterisks indicate significant differences compared with the negative control (*P* value<0.05, Student's *t*-test).

A conserved TACCTACC motif is shared by the regions of the promoters of the seven genes shown to be bound by AtMYB12 (Fig. 2d). This matches the previously reported binding motif for AtMYB12 in *Arabidopsis*^8^,^9^. ChIP-qPCR scanning of the promoters of *ENO*, *DAHPS* and *CHS1* in AtMYB12 tomato fruit showed significant binding of AtMYB12 around this binding motif (Fig. 3a), Promoters of a number of other genes of primary metabolism that showed elevated transcript levels in *AtMYB12* tomatoes compared with controls, were enriched in ChIP of AtMYB12 fruit compared with input DNA (*PGLS, Rpi, ALDO, SUS1, DHQD, SHD* and *PAT*) (Fig. 2b). These may represent additional direct binding targets of AtMYB12, although the sensitivity of the ChIP protocol was not sufficient to establish these as direct interactions with statistical significance. The binding and increased transcript levels of *DAHPS* and *ENO* in response to AtMYB12 imply that these genes are direct targets of AtMYB12 as has been shown for *CHS*, *CHI*, *F3H* and *FLS*^10^.

The MYB12 antibody detected tomato proteins of similar size to AtMYB12 in control tomatoes (Supplementary Fig. 1a). These correspond to the functionally homologous protein in tomato, SlMYB12 (refs 3, 11, 12), as established by immuno-detection of SlMYB12 following expression in *Escherichia coli* (Supplementary Fig. 1b). Since SlMYB12 is not expressed in tomato flesh^12^, flesh offered a robust control on which to establish enrichment in ChIP experiments (Fig. 3b). ChIP-qPCR that scanned binding of SlMYB12 across the promoters of *ENO*, *DAHPS* and *CHS1* in peel of WT tomatoes, showed that SlMYB12 binds to the same DNA motifs as AtMYB12 (Fig. 3c). Consistent with this result, the expression of *ENO*, *DAHPS*, as well as known targets in flavonol biosynthesis, was induced in the peel of WT fruit (Fig. 3b). These data suggest SlMYB12 shares target genes with its functional homologue, AtMYB12.

### AtMYB12 changes carbon flux in tomato fruit

**Figure 4:**
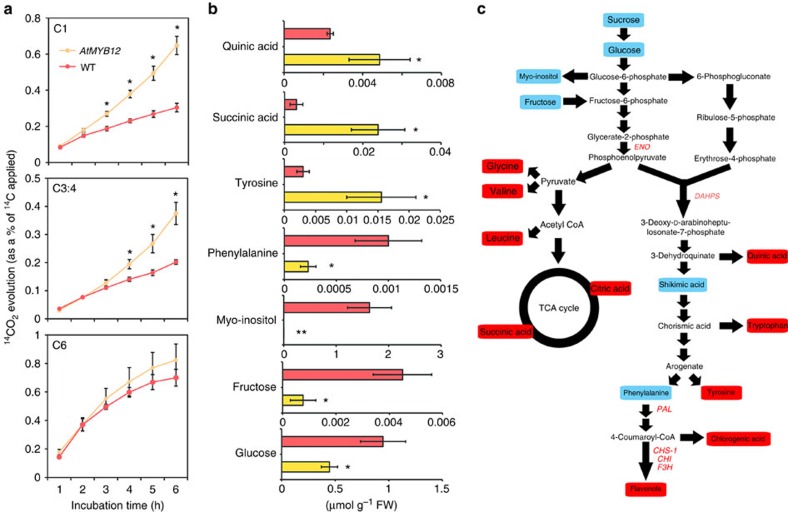
AtMYB12 changes the flux of carbon in tomato fruit. (**a**) Respiratory parameters in fruits of *AtMYB12* and WT. Evolution of ^14^CO_2_ from C1, C3:C4 and C6 position of glucose in pericarp discs of *AtMYB12* and WT tomato fruit 10 days post breaker (10 dpb). Values are means ±s.e.m. of determinations on four independent samples and asterisks indicate values that were significantly different (*P*<0.05) from WT (Student's *t*-test). (**b**) Redistribution of ^13^C label following incubation of *AtMYB12* (yellow) and WT (red) tomato fruits (10 dpb). The absolute isotope redistribution (μmol g^−1^ FW) is shown after an incubation period of 4 h with [U-^13^C] glucose. Values are means ±s.e. of determinations on four independent samples; and asterisks indicate values that were significantly different *(*P*<0.05) from WT (Student's *t*-test). (**c**) Pathway scheme summarizing the metabolic changes in *AtMYB12* tomato compared with WT fruit. Data from RNA-seq, ChIP-qPCR, isotope feeding experiments and metabolomic analyses are summarized. Metabolites which changed significantly (*P*<0.05, Student's *t*-test) in *AtMYB12* tomatoes compared with WT are highlighted in red (for increased) and blue (for decreased). Black arrows represent a route rather than a single metabolic reaction and thus may be comprised of multiple reactions. Genes highlighted in red are direct targets of AtMYB12 as revealed by RNA-seq and ChIP-qPCR data.

Previous studies have shown that *ENO* and *DAHPS* are key genes linking primary metabolism to the specialized metabolism of aromatic amino acids^6^,^13^. Activation of these genes has been shown to enhance primary metabolism and the synthesis of aromatic amino acids and so to increase the supply of precursors for secondary metabolism^6^,^13^. To assess the effect of induction of expression of these genes on respiration rate, we fed pericarp discs at 10 days post breaker (10 dpb) from both *AtMYB12* and WT tomatoes with [1-^14^C] Glc, [3:4-^14^C] Glc, or [6-^14^C] Glc for 6 h. During this period, *AtMYB12* tomatoes released greater amounts of ^14^CO_2_ from all labelling positions, indicating greater flux through glycolysis, the pentose phosphate pathway and the TCA cycle (Fig. 4a)^14^. Calculations of the relative release from the various positionally labelled glucoses^15^ were indicative of a co-ordinated upregulation of glycolysis and the OPPP, rather than a preferential upregulation of one or the other pathway (Supplementary Table 1).

When both *AtMYB12* and control fruit were incubated with uniformly labelled [U-^14^C] Glc for 4 h, there were increases in total uptake, as well as increased label incorporation into organic acids and amino acids in *AtMYB12* tomatoes compared with control tomatoes (Supplementary Table 2). Redistribution of ^13^C label following [^13^C] Glc feeding to pericarp discs also showed that in *AtMYB12* tomatoes, there were significantly higher amounts of the products of the shikimate pathway and the TCA cycle, whereas label in sugars, the major substrates for glycolysis and the oxidative pentose phosphate pathway, were reduced significantly compared with WT tomatoes (Fig. 4b,c). Together, these data indicate that there is greater flux through glycolysis, the pentose phosphate pathway and the TCA cycle in *AtMYB12* tomatoes than in control tomatoes. As a result, the basic sugar and amino acid contents of *AtMYB12* tomatoes were significantly different from WT fruit. There was a significant reduction in the content of the major sugars in *AtMYB12* tomatoes compared with controls (Supplementary Fig. 2) and the contents of aromatic amino acids (except phenylalanine) were significantly higher than in WT fruit (Supplementary Figs 2 and 3). Greater carbon flux through glycolysis, the pentose phosphate pathway and the TCA cycle in *AtMYB12* tomatoes, would provide additional ATP, reducing power and carbon from primary metabolism to drive the biosynthesis of aromatic amino acids and secondary metabolic pathways dependent on these precursors (Fig. 4c). Unlike other TFs, which have been reported to activate the transcription of only key target genes of secondary metabolism, AtMYB12 appears to reprogram primary metabolism, driving carbon flux towards aromatic amino acid biosynthesis, in addition to activating the genes of the flavonoid biosynthetic pathway.

### AtMYB12 enhances flavonoid production induced by other TFs

**Figure 5:**
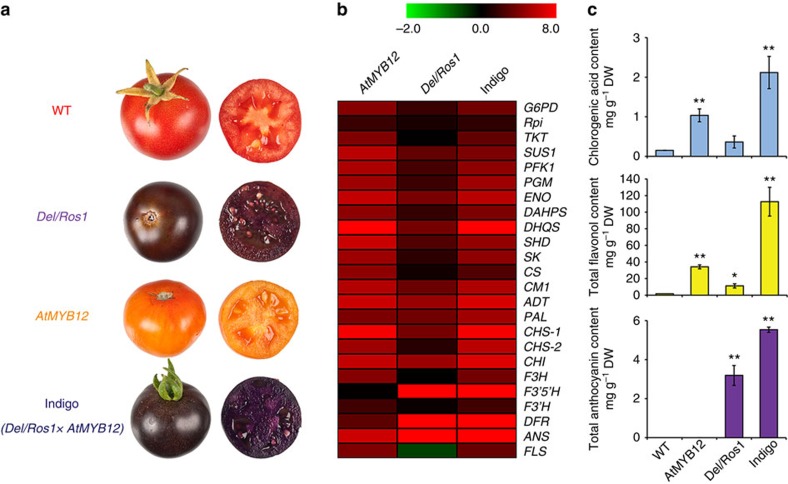
Co-expression of *AtMYB12* with other transcription factors in tomato fruit enhances phenylpropanoid production. (**a**) Phenotypes of WT, *AtMYB12*, *Del/Ros1* and Indigo (*Del/Ros1* × *AtMYB12*) tomato fruit. Pictures were taken at seven days after breaker. (**b**) RT-qPCR data indicated that AtMYB12 activates expression of genes encoding enzymes of primary metabolism and first stages of flavonoid biosynthesis, while Del/Ros1 mainly activates genes encoding enzymes late in flavonoid biosynthesis. For Indigo tomato, however, all genes encoding enzymes of primary and secondary metabolism were highly upregulated. The heat map compares transcript levels in the different tomato lines to those in WT fruit. Absolute values are scaled by log2. The details of all genes are explained in Supplementary Table 7. (**c**) The contents of the major phenylpropanoids (CGA, flavonols and anthocyanins) were significantly increased in Indigo tomatoes compared with other tomato lines. Asterisks indicate values that were significantly different *(*P*<0.05), **(*P*<0.01) from WT (Student's *t*-test). Error bars show s.e.m. (*n*=3).

To explore further the potential of *AtMYB12* for engineering phenylpropanoid metabolism, we crossed *AtMYB12* tomato to the purple, *Del/Ros1* tomato line, which accumulates high levels of anthocyanins in fruit^2^. The hybrid tomato showed striking, intense blue-purple colour. The change in hue was the result of co-pigmentation of anthocyanins by high levels of flavonols, throughout the fruit. We termed this phenotype Indigo (Fig. 5a). In contrast to AtMYB12, which activates genes encoding enzymes of both primary metabolism and general phenylpropanoid metabolism (Fig. 5b), Del and Ros1 enhanced anthocyanin biosynthesis principally by activating the expression of anthocyanin biosynthetic genes (Fig. 5b). Del and Ros1 did not appear to activate expression of genes in primary metabolic pathways significantly and, unlike *AtMYB12* tomatoes, the content of aromatic amino acids did not increase in purple *Del/Ros1* fruit compared with WT fruit (Supplementary Fig. 3).

Overexpression of *AtMYB12* together with *Delila* and *Rosea1* activated all the genes encoding enzymes of primary metabolism through to flavonoid biosynthesis (Fig. 5b). Compared with parental lines, Indigo tomato fruit had even greater contents of chlorogenic acid (CGA) (twofold more than *AtMYB12* tomatoes), flavonols (3-fold more than *AtMYB12* tomatoes) and anthocyanins (almost twofold more than purple *Del/Ros1* tomatoes) (Fig. 5c and Supplementary Table 3). Compared with Del and Ros1, we suggest that AtMYB12 has a greater capacity to increase the production of different phenylpropanoids in tomato fruit through its impact on the supply of amino acids, ATP and reducing power in fruit in addition to its ability to activate flavonoid biosynthesis. We also checked the content of malonyl-CoA, another key substrate of flavonoid biosynthesis^16^, but despite our method being able to detect this metabolite in *Arabidopsis*, malonyl-CoA could not be detected in any tomato fruit samples even following solid phase extraction pre-purification. We were, however, able to measure the levels of acetyl-CoA, the direct precursor of malonyl-CoA^16^ and found no significant differences in levels between different lines (Supplementary Fig. 4). These data are not consistent with any depletion of the malonyl-CoA pool limiting flavonoid biosynthesis and indicate that AtMYB12 enhances flavonoid biosynthesis in plants, mainly through effects on phenylalanine biosynthesis.

### AtMYB12 can enhance production of novel phenylpropanoids

**Figure 6:**
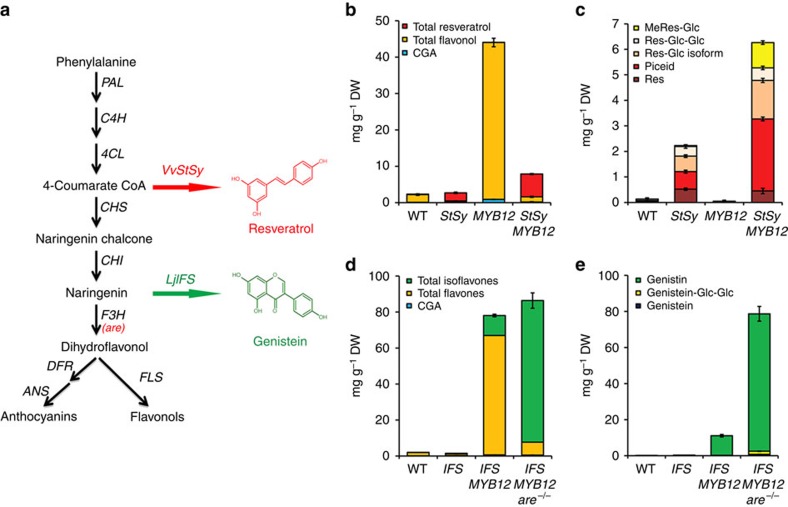
Co-expression of *AtMYB12* with other structural genes in tomato fruit enhances novel phenylpropanoid production. (**a**) Schematic representation of resveratrol and genistein biosynthesis. Both pathways arise from flavonoid biosynthesis (in black) by the addition of *VvStSy* and *LjIFS* respectively. After synthesis, both resveratrol and genistein are glycosylated to form piceid and genistin. The naturally occurring *are* mutant is deficient in F3H activity. (**b**) The contents of CGA, total flavonols and total resveratrol compounds in different genotypes. Fruit were harvested 10 days after breaker. Error bars show s.e.m. (*n*=3). (**c**) The detailed contents of resveratrol (Res), piceid, resveratrol-glycoside isoform (Res-Glc Isoform), resveratrol-di-glycosides (Res-Glc-Glc) and methylated resveratrol-glycosides (MeRes-Glc) in different genotypes. Error bars show s.e.m. (*n*=3). (**d**) The contents of CGA, total flavonols and total isoflavones in different genotypes. Fruit were harvested 10 days after breaker. Error bars show s.e.m. (*n*=3). (**e**) The detailed contents of genistein, genistin and genistein-di-glucosides (genistein-Glc-Glc) in different genotypes. Error bars show s.e.m. (*n*=3).

Our data suggested that AtMYB12 could be used to enhance the production of specialty phenylpropanoid compounds in tomato. Resveratrol (3,5,4'-tri-hydroxystilbene) is a natural phytoalexin and has been reported to extend the lifespan of several model organisms^17^. The therapeutic potential of resveratrol has been established both in *vivo* and *in vitro*^18^. Overexpression of a gene encoding stilbene synthase (*StSy*) from grape has been shown to result in accumulation of resveratrol in tomato fruit up to about 0.5 mg g^−1^ DW (all forms of resveratrol) (Fig. 6a)^19^. We crossed the *E8:AtMYB12* tomato to an original *35S:VvStSy* tomato line to select tomato fruit with different genotypes. LC-MS data confirmed that the original *35S:VvStSy* tomato produces ∼0.5 mg g^−1^ DW trans-resveratrol in fruit (Fig. 6b,c and Supplementary Table 4)^19^. However, after co-expression with *AtMYB12*, the production of resveratrol and resveratrol derivatives reached between 5 and 6 mg g^−1^ DW, some 100-fold higher than levels found in red grapes (Fig. 6b,c and Supplementary Table 5) due primarily to a 4–5-fold increase in polydatin (monoglycosylated resveratrol). Interestingly, the high levels of flavonols observed in *AtMYB12* tomatoes, were not observed in *AtMYB12 StSy* tomatoes suggesting that stilbene synthase competes very effectively with chalcone synthase to redirect synthesis towards stilbenes and away from flavonoids (Fig. 6b). Significant amounts (∼1 mg g^−1^ DW) of a mono-methylated resveratrol were detected in *AtMYB12 StSy* tomatoes (Fig. 6c), which are of interest because methylated stilbenes have been reported to have greater bioactivity than resveratrol, as assayed in several disease models^20^,^21^.

Genistein is the major isoflavone found in soybean. Genistein has been suggested to play a role in the prevention of steroid hormone-related cancers, particularly breast cancer, due to the significantly lower incidence of steroid-hormone responsive cancers in Asian communities consuming a high-soy diet (30% lower incidence of prostate and breast cancers [ http://www.cdc.gov/cancer/prostate/statistics/race.htm]). Genistein is thought to act as a phytoestrogen by binding to the human oestrogen receptor^22^. To engineer isoflavone biosynthesis in tomato, we overexpressed an isoflavone synthase gene (*LjIFS*) from *Lotus japonicus*^23^ in tomato (*Solanum lycopersicum* cv. MicroTom) under the control of the fruit-specific E8 promoter (Fig. 6a). Expression of *LjIFS* under the control of the E8 promoter resulted in low levels of the glycosylated isoflavone, genistin, in tomato fruit (Supplementary Fig. 5), although these were 2–10-fold higher than the genistin levels reported following expression of IFS from soybean driven by the CaMV 35S promoter in tomato^24^. To determine whether we could enhance isoflavone production yet further, we crossed the *AtMYB12* tomato line with the *are* (anthocyanin reduced) mutant, deficient in flavanone 3-hydroxylase activity (F3H) (Fig. 6a)^25^,^26^ to make *AtMYB12 are*^−*/*−^. Compared with *AtMYB12* tomato, *AtMYB12 are*^−*/*−^ tomato fruit had only 30% flavonols in their fruit (Supplementary Fig. 6). We chose the *LjIFS* line which had the highest genistin content in fruit (Supplementary Fig. 5) and crossed it to an *AtMYB12 are*^−*/*−^ tomato line and selected different genotypes in the F2 generation (Fig. 6a and Supplementary Fig. 7). In fruit expressing *LjIFS* only, the content of genistin, the only isoflavonoid detected, was ∼0.3 mg g^−1^ DW. After the introduction of *AtMYB12*, the genistin content was significantly increased to about 11 mg g^−1^ DW. In lines homozygous for the *are*^−*/*−^ mutation, the total amount of isoflavones increased to reach about 78 mg g^−1^ DW, some 100-fold higher than genistin levels in soy products such as tofu and natto (Fig. 6d,e and Supplementary Table 5). This was due to a significant reduction in flavonol production achieved by reducing F3H activity using the *are*^−*/*−^ mutation (Fig. 6a,d and Supplementary Table 5). Because IFS competes directly with F3H for substrate (naringenin; Fig. 6a)^27^, a reduction in F3H activity significantly increased the production of isoflavones in tomato. In contrast, when *are*^−*/*−^ was introduced into *AtMYB12 StSy* tomatoes, the content of resveratrol did not change significantly (Supplementary Table 4), presumably due to the strongly competitive activity of stilbene synthase relative to that of chalcone synthase, which reduces flux to flavonols.

## Discussion

For both resveratrol and genistin production in tomato, co-expression of *AtMYB12* substantially increased the production of these specialty phenylpropanoids. We suggest that the impact of AtMYB12 on metabolic engineering depends not only on transcriptional activation of genes encoding enzymes of secondary metabolism but also its ability to reprogram carbon flux towards aromatic amino acid biosynthesis to increase the supply of substrate as well as energy and reducing power for phenylpropanoid metabolism. Compared with induced expression of key biosynthetic genes, reprogramming carbon flux represents a powerful way to enhance the levels of target metabolites in plants^28^.

We propose that the effects of AtMYB12 on flux and primary metabolism are not only because of the strong sink effects of the phenylpropanoid biosynthetic pathway. Instead we suggest that they are an effect of increasing the supply of carbon, reducing power and ATP from primary metabolism into the shikimate pathway and that as a result, the content of other aromatic amino acids (Trp and Tyr) increase significantly in *AtMYB12* tomatoes, while the content of the major sugars decreases significantly (Supplementary Figs 2 and 3). In contrast, TFs like Delila and Rosea 1 provide only a strong sink for anthocyanin biosynthesis without upregulating primary metabolism^29^ and the content of other aromatic amino acids does not increase (or even decreases) in *Del/Ros1* tomatoes (Supplementary Fig. 3). We propose that the result of these different effects of AtMYB12 (controlling both supply and demand) and Del/Ros1 (controlling demand only) is the reason why net accumulation of flavonols driven by AtMYB12 is always greater (by at least threefold) than the highest levels of anthocyanins achieved using Del and Ros1 (Fig. 5c and Supplementary Table 3). We cannot formally exclude the possibility that the large effects of AtMYB12 are due solely to the increased sink strength of the phenylpropanoid pathway given that inhibitor studies or RNAi of PAL in developing fruit would be very difficult to interpret. That said, we firmly believe that the combination of source-sink and upstream activation effects increase the content of aromatic amino acids in *AtMYB12* tomato, making it an excellent tool for engineering metabolites derived from tyrosine and tryptophan as well as phenylpropanoids derived from phenylalanine.

Our data show that AtMYB12 recognizes the same suite of target genes as its functional homologue, SlMYB12, in tomato. This is supported by genome-wide transcriptome analysis of tomato fruit with reduced SlMYB12 activity as a result of the *y* mutation^11^. Flavonoid accumulation in the peel of tomato is controlled by an SlMYB12-regulated transcriptional network^11^. The homologous TF to AtMYB12 in maize, P1, has also been reported to modify the expression of a large number of genes including some encoding enzymes of glycolysis^30^, although it is not clear whether any genes of primary metabolism are direct targets of P1.

It may be that some TFs targeting secondary metabolic pathways have additional targets involved in primary metabolism to ensure that the demands of specialised secondary metabolism can be met by adequate supplies of precursors, energy and reducing power. Such regulatory targets do not exist for all TFs in a regulatory network, as shown by comparison of AtMYB12 to the effects of Del and Ros1 on transcript levels in tomato fruit (Fig. 5a). Such regulatory input is likely only a small component of the networks defining coarse control of primary metabolism, and is unlikely to be observed during the analysis of the effects of mutations affecting activity of specific TFs. Effects on target genes in primary metabolism are more likely to be observed following ectopic or overexpression of a TF, but this does not imply that they are an artefact of high-level expression. It is possible that such targets can be identified only by ectopic or high level expression of TFs, and are more likely to be observed when expression is driven by an inducible promoter, such as the E8 promoter in tomato fruit. When AtMYB12 expression is driven by a constitutive promoter such as the CaMV 35S promoter, its effects on flavonol accumulation are more modest than when driven by the E8 promoter in fruit^3^,^31^,^32^. This is likely because of metabolite repression of AtMYB12-induced activation of primary metabolism in actively photosynthesising tissues, to maintain growth and development since we observed reduced growth of tobacco plants expressing *AtMYB12* under the control of the 35S promoter^3^. Our characterisation of the targets of AtMYB12 refines our understanding of metabolic control in eukaryotes, where the contribution of transcriptional control of central metabolism (supply) has been considered to be modest compared with transcriptional control of demand^28^,^33^,^34^,^35^. Most studies that dismiss the contribution of control of central/primary metabolism have been founded on the effects of loss of function of TFs, and perhaps more importance would be attached to the transcriptional control of supply by incorporating studies of the effects of overexpression of regulators as well.

The health benefits of phenylpropanoids make them important targets for metabolic engineering. For many plant secondary metabolites, use of plants for engineering high level production remains an effective option^36^. Enrichment of bioactives in tomato could be particularly useful for the comparison of the nutritional properties of different bioactives in food. For example, while there are many publications reporting beneficial effects of consumption of phytoestrogenic isoflavones in combatting the symptoms of menopause and in protection against breast and prostate cancer, there are others that suggest that dietary isoflavones can be detrimental to health^37^, especially for some population groups. The high genistin tomatoes we have engineered could be assessed for their dietary impact on a range of disease models, including models for breast and prostate cancer, to define more precisely the effects of dietary isoflavones on these diseases.

We have shown that AtMYB12 can be used as a general tool to engineer appreciable levels of phenylpropanoids in tomato. By direct activation of plastidial *ENO* and *DAHPS*, as well as genes of secondary metabolism, AtMYB12 can reprogram metabolism towards the biosynthesis of phenylpropanoids. Co-expression of AtMYB12 with other TFs or structural genes results in the highest yields so far reported for anthocyanins, stilbenes and isoflavones in tomato fruit. Metabolic engineering that combines multi-level transcriptional regulation and pathway rerouting offers an excellent strategy for biofortification of foods, for the production of plant-derived phytochemicals and ingredients, and for establishing materials for comparative nutrition studies. Such comparisons should lead to much clearer understanding of the health benefits of foods rich in specific polyphenolic phytonutrients in the diet, and shed light on their mechanisms of action.

## Methods

### RNA isolation and RNA-sequencing

Both WT and *AtMYB12* MicroTom fruit were tagged at breaker stage and harvested at seven days after breaker. Fruit pericarp was ground into fine powder using liquid Nitrogen. RNA isolation was performed using an RNeasy plant mini kit (Qiagen, http://www.qiagen.com/). First-strand cDNA was synthesized from 2 μg of total RNA using oligo(dT) and SuperScript III (Invitrogen, http://www.lifetechnologies.com/uk/en/home.html).

RNA-sequencing was undertaken using EXPRSS Tag-seq following library construction^38^. Libraries were sequenced using Illumina Genome Analyzer II.

The Illumina sequence library was quality filtered using FASTX Toolkit 0.0.13 with parameters -q20 and -p50 (http://hannonlab.cshl.edu/fastx_toolkit/index.html). Reads containing ‘N' were discarded. The libraries were separated on the basis of perfect matches to the barcode. The sub-library was artefact filtered using FASTX-toolkit. The quality filtered library was aligned to the *Solanum lycopersicum* cDNA sequences (ITAG2.3 ftp://ftp.solgenomics.net/tomato_genome/ annotation/ITAG2.3_release/ITAG2.3_cdna.fasta) using Bowtie version 0.12.8 (ref. 39).

Unaligned reads were aligned to the tomato genome sequence (ftp://ftp.solgenomics.net/ tomato_genome/annotation/ITAG2.3_release/ITAG2.3_genomic.fasta) using Bowtie version 0.12.8. Tag to gene association was carried out using the following considerations: reads aligning to each gene limits were assigned to that gene, reads aligning to genes with overlapping gene limits were split equally between them, reads aligning to more than 10 genes were discarded. Differential expression analysis was performed using the R statistical language version 2.11.1 with the Bioconductor package^40^, edgeR version 1.6.15 (ref. 41) with the exact negative binomial test using tagwise dispersions. The RNA-seq raw data for this work have been deposited in the GEO database under accession ID **GSE61014**.

### Protein expression and detection

The cDNA of *AtMYB12* (At2g47460), *SlMYB12* (Solyc01g079620) and *AtMYB75* (At1g56650) were cloned into Gateway pDEST17 vector (Invitrogen, 11803-012), which has an N-terminal 6 x His tag. Vectors for expression in *E.coli* were transferred to expression host Rosetta II using heat shock method. Bacterial cultures were grown overnight at 37 °C in LB medium containing the required antibiotics (chloramphenicol+vector specific) and were sub-cultured to an OD_600_=0.8. Uninduced culture (1 ml) was collected. Protein expression was induced by addition of 0.4–1 mM IPTG and the culture was grown for 4 h at 37 °C. After the required period of growth, 1 ml of induced culture, as well as uninduced culture, were centrifuged at 12,000 r.p.m., suspended in 10% SDS for SDS–polyacrylamide gel analysis. To detect recombinant proteins, Monoclonal Anti-polyHistidine−Peroxidase antibody produced in mouse (4 μg of antibody per gel, Sigma, A7058) was used.

### Chromatin immunoprecipitation

ChIP experiments were done using WT and *AtMYB12* tomato fruit at 3–4 days after breaker (when the expression of *AtMYB12* is strongly induced). To cross-link protein and DNA, fruit were sliced into small pieces and immersed in crosslinking buffer (0.4 M Sucrose, 10 mM Tris-HCl pH 8.0, 0.1% β-mercaptoethanol, 100 μM PMSF, 1% formaldehyde and 1x protease inhibitor cocktail (Roche, http://www.roche.com/index.htm) and vacuum infiltrated two times, for 10 min each time. To terminate crosslinking, glycine was added to a final concentration of 0.125 M and samples were vacuum infiltrated for an additional 5 min. After crosslinking, samples were washed in ice-cold water and ground in liquid nitrogen. Chromatin isolation was performed using Honda buffer^42^. About 3–6 g of sample tissue were re-suspended with 25 ml Honda buffer on ice for 5 min and filtered through two layers of Miracloth. Pellets were collected after centrifugation at 2,000 × *g* at 4 °C for 10 min and resuspended again with 1 ml Honda buffer, repeating the centrifugation and resuspension at least three times, until the pellets were almost transparent. Pellets could be stored at −80 °C for 2 weeks before further treatment. Chromatin was isolated by resuspending each pellet in 320 μl Nuclei Lysis Buffer (50 mM Tris-HCl pH 8, 10 mM EDTA, 1% SDS, 1 mM PMSF and 1 × Protease Inhibitors) and ChIP was performed using anti-AtMYB12 polyclonal antibody (prepared in rabbit against peptide sequence: CLLDGDDEATIGNSN; GenScript USA Inc) (4 μg of antibody per immunoprecipitation). DNA was recovered after immunoprecipitation^43^.

ChIP-qPCR was performed on three independent replicates with appropriate primers (Supplementary Table 5). The *ACTIN* gene (Solyc11g005330) from tomato was used as the internal control for the ChIP experiments. Data were represented as the ratio of (target genes/*ACTIN* in ChIPed DNA) to (target genes/*ACTIN* in Input DNA).

ChIP-sequencing (ChIP-seq) was undertaken using one sample of *AtMYB12* tomato ChIPed DNA and its input as control. Library construction was done using an Illumina ChIP-seq Sample Prep Kit following the manufacturer's instructions and sequencing was undertaken on Illumina's HiSeq platform (Beijing Genomics Institute (BGI), China). Raw data from ChIP-seq have been deposited in the GEO database under accession ID **GSE62462**.

Filtered ChIP-seq data were aligned to the tomato (*Solanum lycopersicum*) genome sequence (ftp://ftp.solgenomics.net/tomato_genome/annotation/ITAG2.3_release/ITAG2.3_genomic.fasta) using Bowtie^39^. Peak discovery was performed using MACS (BGI, China)^44^. The output files were submitted to the genome browser of Tomato Epigenome Database (http://ted.bti.cornell.edu/epigenome/)^45^ to visualize the peaks in the tomato genome.

### Feeding experiments with ^14^C- and ^13^C-labelled glucose

Pericarp discs of 10 mm diameter were excised from mature fruits and incubated in 37 kBq [U-^14^C]- glucose (specific activity 8.11 MBq mmol^−1^), 2.32 kBq ml-1 of 1-[^14^C]-, 3,4-[^14^C]- or 6-[^14^C]- glucose, or 10 mM U-[^13^C]- glucose (purchased from Amersham International and Euriso-Top for radiolabeled and stable isotopes, respectively), eight discs were incubated in Erlenmeyer flasks with 5 ml of 10 mM MES-KOH buffer (pH 6.5). For positionally labelled glucose, flasks were sealed and shaken at 90 r.p.m., at 25 °C. The evolved ^14^CO_2_ was trapped in 0.5 ml of 10% (w/v) KOH which was replaced with a fresh source of KOH every hour and the level of radiolabel determined by liquid scintillation counting. The ratio of ^14^CO_2_ released from 1-[^14^C]-glucose and 6-[^14^C]-glucose is indicative of the relative flux through glycolysis and the oxidative pentose phosphate pathways with higher ratios indicating a preference for the glycolytic route and lower ratios that for the oxidative pentose phosphate pathway. Similarly, the ratio of ^14^CO_2_ released from 1-[^14^C]-glucose and 3,4-[^14^C]-glucose indicates the relative activities of glycolysis and the TCA cycle with smaller ratios indicating a preference for the TCA cycle^15^,^46^. Following 4 h incubation in either U-[^14^C]-glucose or U-[^13^C]-glucose, the pericarp discs were carefully washed in fresh incubation media, dried and snap-frozen in liquid nitrogen, before analysis.

### Evaluation of label redistribution in radiolabelled samples

Pericarp discs were extracted with 80% (v/v) ethanol at 80 °C (1 ml per two discs), re-extracted in two subsequent steps with 50% (v/v) ethanol (1 ml per two discs at each step), the supernatants were combined, dried under an airstream at 40 °C, the ethanol-soluble components were resuspended in 1 ml water and separated into neutral, anionic and basic fraction as the following; a 750 μl aliquot of the ethanol-soluble fraction was loaded onto the combined ion-exchange column and allowed to drain before washing the column with two 1 ml aliquots of double distilled water (ddH_2_O). The elute of these washes was collected in a 5 ml scintillation tube and is termed the neutral fraction. The cationic and anionic columns were then separated and placed in scintillation tube. The cationic column was eluted with two washes of 1 ml 1 M ammonium hydroxide and the eluate was termed the basic fraction. The anionic column was eluted with two washes of 1 ml 4 M formic acid and the eluent was termed the acidic fraction. The acidic fraction corresponds to organic acids and the basic fraction corresponds to amino acids. The ^14^C content of an aliquot of each fraction was determined by scintillation counting. Labelled sucrose levels were determined after 4 h incubation of 200 μl of total neutral fraction with 4 units per ml of hexokinase in 50 mM Tris-HCl, pH 8.0, containing 13.3 mM MgCl_2_ and 3.0 mM ATP at 25 °C. For labelled glucose, 200 μl of neutral fraction were incubated with 1 unit ml^−1^ of glucose oxidase and 32 units ml^−1^ of peroxidase in 0.1 M potassium phosphate buffer, pH 6, for a period of 6 h at 25 °C. After the incubation time, all reactions were stopped by heating at 95 °C for 5 min. The label was separated by ion-exchange chromatography as described above^47^. The ethanol-insoluble fraction was homogenised in ddH_2_O and adjusted to a known volume with ddH_2_O, after a 200 μl aliquot of the ethanol –insoluble sample was taken and combined with 500 μl 200 mM Na-acetate buffer (pH 5.5) containing 10 units of amyloglucosidase and 2 units amylase. The reaction was incubated at 37 °C for 16 h. At the end of the incubation, the digest mixture was buffered to pH 7.8 with 100 mM Tris-HCl and 10 units pronase was added. The reaction was incubated at 37 °C for further 16 h. the reaction mixture was centrifuged at 6,000 × *g*. The entire supernatant was subjected to ion-exchange fractionation as described above. The neutral fraction corresponded to starch, the basic fraction corresponded to protein and the acidic and pellet fractions corresponded to cell wall material. The ^14^C content of all fractions was quantified by scintillation counting^47^. Hexoses in this experiment were fractionated enzymatically based on the rationale that hexose sugars will be converted to their respective hexose phosphates via the action of hexokinase leaving sucrose as the only major component of the neutral fractionation. Specific activities were estimated by dividing the label retained in the phosphoester fraction by the summed carbon in these fractions and this data was used to determine the flux to starch, cell wall, and sucrose^48^ and the summed label in organic acids, amino acids and protein was used to determine the carbohydrate oxidation flux.

### Evaluation of label redistribution in stable-isotope samples

Pericarp discs were extracted in 100% methanol at 70 °C for 15 min. After centrifugation, the resultant supernatant was dried under vacuum, and the residue was derivitized for 120 min at 37 °C (in 50 μl of 20 mg ml^−1^ methoxyamine hydrochloride in pyridine) followed by a 30 min treatment at 37 °C with 50 μl of MSTFA. The GC–MS system used was a gas chromatograph coupled to a time-of-flight mass spectrometer (Pegasus III, Leco). An autosampler system (PAL) injected the samples. Helium was used as carrier gas at a constant flow rate of 2 ml s^−1^ and gas chromatography was performed on a 30 m DB-35 column. The injection temperature was 230 °C and the transfer line and ion source were set to 250 °C. The initial temperature of the oven (85 °C) increased at a rate of 15 °C min^−1^ up to a final temperature of 360 °C. After a solvent delay of 180 s mass spectra were recorded at 20 scans s^−1^ with *m/z* 70–600 scanning range. Chromatograms and mass spectra were evaluated by using Chroma TOF 1.0 (Leco) and TagFinder 4.0 software^49^, for calculation of ^13^C enrichment, a peak intensity matrix containing all available mass isotopomers of characteristic mass fragments that represented the primary metabolites was generated by TagFinder. This matrix was processed using CORRECTOR software tool (http://www-en.mpimp-golm.mpg.de/03-research/researchGroups/01-dept1/Root_Metabolism/smp/CORRECTOR/index.html). Using this processing tool the sum of mass isotopomer intensities and the ^13^C enrichment of mass fragments was then calculated^15^.

### Plasmid construction and tomato transformation

The full length sequence of *LjIFS* cDNA (GB ID: AB024931) was first amplified by PCR using Gateway compatible primers and inserted into pDNOR207 to make pENTR207-LjIFS. The *LjIFS* cDNA was then inserted into the Gateway cassette of plasmid pSLJ.E8.1500 (ref. 2) to make pE8:IFS. The pE8:IFS plasmid was transferred to *Agrobacterium tumefaciens* strain LBA4404 by triparental mating. Tomato transformation was undertaken using cotyledons^3^.

### Analysis and identification of phenylpropanoids

For LC-MS analysis of phenylpropanoid compounds, tomato fruit were harvested 7 days after breaker. The whole fruit pericarp was freeze dried and ground into a fine powder. An amount of 1 g of dry powder was extracted with 25 ml 80% MeOH at 4 °C under agitation, overnight. The sample was then centrifuged at 3,000 × *g* at 4 °C for 15 min and the supernatant was taken. The pellet was extracted again with 25 ml 80% MeOH at 4 °C for another 2 h. The supernatant was combined and further diluted 10 times with 80% MeOH. The samples were filtered through a 0.45 μm filter before injection. For each line, three biological replicates were analysed.

For flavonoid and isoflavone analysis, the samples were run on a Surveyor high-performance liquid chromatography (HPLC) system (Thermo) attached to a DecaXPplus ion trap MS (Thermo). Separation was on a 100 × 2.1 mm 2.6 μ Kinetex XB-C18 column (Phenomenex) using the following gradient of methanol versus 0.1% formic acid in water, run at 200  μl min^−1^ and 30 °C: 0 min, 2% MeOH; 24 min, 38% MeOH; 30 min, 70% MeOH; 33.6 min, 70% MeOH, 34.2 min, 2% MeOH and 43.2 min, 2% MeOH. Data were collected for 34 min. UV-visible absorbance was collected with spectra from 200–600 nm from which chromatograms could be extracted at any wavelength, and a specific channel of 260 nm (bandwidth 9 nm), and positive electrospray MS spectra from *m/z* 150–2,000. The instrument also collected data-dependent MS2 spectra at an isolation width of *m/z* 4.0 and 35% collision energy, using dynamic exclusion to maximize the number of ions selected for fragmentation. Spray chamber conditions were 50 units sheath gas, 350 °C capillary temperature, and a spray voltage of 3.8 kV. Sheath gas was supplied via the aux gas line and the entire flow from the LC passed through diode array to the mass spec ionization chamber without teeing.

Resveratrols were measured using a Surveyor HPLC system attached to a DecaXPplus MS (both Thermo). Separation was on a 100 × 2 mm 3 μm Luna C18(2) column (Phenomenex) running the following gradient of acetonitrile (ACN) versus 0.1% formic acid in water at 30 °C and 250 μl min^−1^: 0 min, 2% ACN; 30 min, 70% ACN; 30.5 min, 2% ACN and 38 min, 2% ACN. resveratrol and polydatin were detected by UV absorbance at 306 nm (9 nm bandwidth) and by selected reaction monitoring in positive mode electrospray MS. The mass spec was set up to collect full spectra from *m/z* 150–1,000 and also targeted MS2 of precursor ion 229.0 at 40% collision energy and an isolation width of *m/z* 4.0, and precursor 391.1 at 40% collision energy and an isolation width of *m/z* 3.0. Spray chamber conditions were 50 units sheath gas, 5 units aux gas, 350 °C capillary temperature, and a spray voltage of 3.8 kV using a steel needle kit.

### Amino acid measurements

Fruit were harvested seven days after breaker and 50 mg of freeze dried fruit pericarp was extracted with 1 ml 75% EtOH. The extracts were centrifuged for 10 min at 13,000 × g at 4 °C. The supernatant was taken and the pellet was extracted two more times with 1 ml 75% EtOH. The supernatants (3 ml in total) were combined and evaporated to dryness in the evaporator. The dried residue was then dissolved in 10 ml of 0.02 M HCl (pH 1.7–1.8) and filtered through a cellulose acetate filter (0.45 μm). Derivatizing was done using an AccQ-Tag Chemistry Kit from Waters (http://www.waters.com/). Samples (15 μl) were derivatized to final volume of 100 μl and 10 μl were injected for measurement. For amino acid measurements, samples were measured using Agilent 100 LC-MS system. Separation was on a 100 × 2.1 mm Kinetex XB-C18 Column (Phenomenex) running the following gradient of acetonitrile (can) versus 0.1% formic acid in water at 30 °C and 300 μl min^−1^: 0 min, 1% ACN; 2 min, 1% ACN; 30 min, 25% ACN; 33.5 min, 90% CAN; 34.5 min, 90% ACN, 35 min, 1% ACN and 45 min, 1% ACN. Amino acids were detected by UV absorbance at 260 nm (9 nm bandwidth) and by selected reaction monitoring in positive mode electrospray MS of the m/z 150–1,000 range. The MS working conditions were: Nebulise pressure 25 psi; drying gas flow 11.5 l min^−1^ at 350 °C; spray voltage 4kV.

### Acetyl-CoA and malonyl-CoA measurement by LC-MS/MS

Extraction of acetyl-CoA and malonyl-CoA was performed by a method modified from that described by Perera et al^50^. The ground frozen materials (100 mg) were extracted in 500 μl of pre-cooled 1% TFA water with homogenization for 2 min at 25 l s^−1^. Supernatant was obtained after centrifugation at 11,000 × *g* at 4 °C for 10 min, and 500 μl of diethylether was added. The water fraction obtained after vortexing and centrifugation at 11,000 × *g* at 4 °C for 10 min, was evaporated without heating. The pellet was re-suspended by mixing with 200 μl of 10 mM ammonium formate solution at 4 °C. Resuspended fractions were loaded onto an Strata-X 33 u Polymeric Reversed Phase column (60 mg, 3 ml) (Phenomenex) and prewashed/equilibrated consecutively with 3 ml of methanol and 10 mM ammonium formate. CoA fractions were eluted in the first fraction with 10 mM ammonium formate, and evaporated without heating. The dried elute was dissolved in 100 μl of 10 mM ammonium formate. After centrifugation, supernatant was subjected to liquid chromatography (LC)–tandem MS (MS/MS) in negative ion detection mode using an HPLC Surveyor System coupled to an LTQ Linear ion trap (IT) ESI-MS (Thermo Finnigan). HPLC separation was performed with Luna C18 column, (1.0 × 50 mm, 3 μm particle size; Phenomenex) at a flow rate of 100 μl min^−1^ of 10 mM ammonium formate as solvent A and MeOH as solvent B with following linear gradient from 0% B to 0% B for 5 min, from 0% B to 5% B for 5 min, and to 100% B for 4 min. The specific fragment mass peaks (malonyl-CoA and acetyl-CoA, 347 and 303 *m/z*, positive ion detection) derived from MS/MS fragmentation (collision energy, 65 eV) of the molecular parental ion peak (malonyl-CoA and acetyl-CoA, 854 and 810 *m/z*) were profiled. Chromatograms were processed and selected peak areas were quantified using Quan Browser of Xcalibur software (Thermo Finnigan).

### Statistics

Unless specifically described, paired or unpaired, two-tailed Student's *t*-tests were used to compare group differences throughout this study.

## Additional information

**Accession codes**: Sequence data used in this study have been deposited in the GEO database under accession ID GSE61014 and GSE62462.

**How to cite this article:** Zhang, Y. *et al.* Multi-level engineering facilitates the production of phenylpropanoid compounds in tomato. *Nat. Commun.* 6:8635 doi: 10.1038/ncomms9635 (2015).

## Supplementary Material

Supplementary Information Supplementary Figures 1-7 and Supplementary Tables 1-7

Supplementary Data Differentially expressed genes in *AtMYB12* tomato fruit

## Figures and Tables

**Figure 1:**
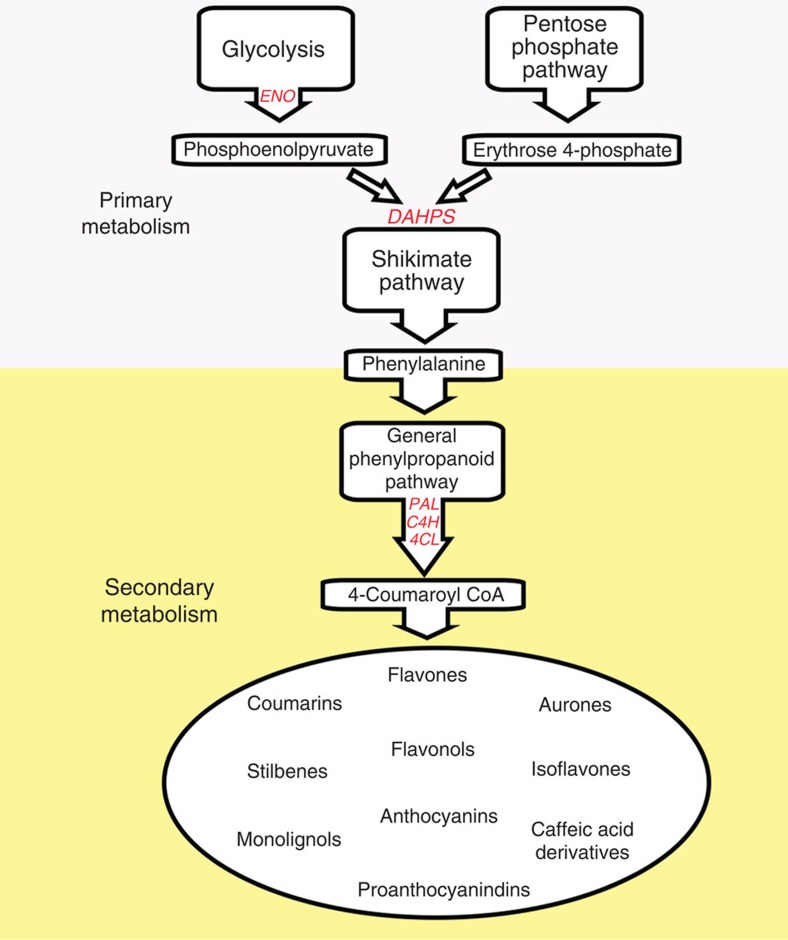
Schematic representation of the phenylpropanoid pathway in plants and its relationships to primary metabolic pathways. Important primary and secondary metabolic genes are highlighted in red. *DAHPS*, 3-deoxy-D-arabino-heptulosonate 7-phosphate synthase; *ENO*, plastidial enolase; *PAL,* phenylalanine ammonia lyase; *C4H*, cinnamate 4-hydroxylase; *4CL*, 4-coumaroyl CoA ligase.
