# Supplementary material for: Multi-level engineering facilitates the production of phenylpropanoid compounds in tomato
Source: Nat Commun. 2015 Oct 26;6:8635. doi: 10.1038/ncomms9635 (PMC4639801; doi:10.1038/ncomms9635)
Supplement: Supplementary Information — Supplementary Figures 1-7 and Supplementary Tables 1-7 [file ncomms9635-s1.pdf]

Supplementary Figures

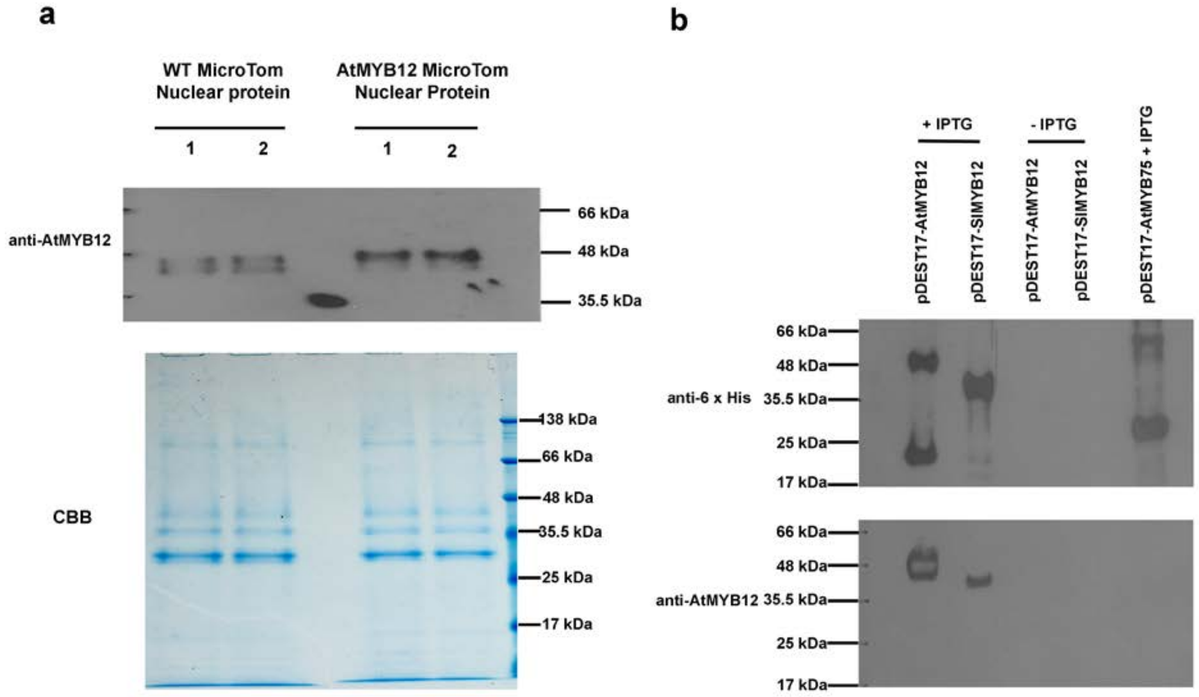

Supplementary Figure 1. AtMYB12 antibody detects both *Arabidopsis* and tomato MYB12 protein. (a) AtMYB12 antibody detects both SlMYB12 and AtMYB12 in tomato fruit. Both WT and *AtMYB12* tomato fruit were harvested three days after breaker. Nuclei from both fruit were isolated and nuclear protein extracts were assessed by western blot. Coomassie brilliant blue staining (CBB) showed equal loading of proteins from WT and *AtMYB12* tomato extracts. (**b**) AtMYB12 antibody detects both SlMYB12 and AtMYB12, but not AtMYB75 recombinant proteins. *AtMYB12*, *SlMYB12* and *AtMYB75* cDNAs were cloned into the pDEST17 plasmid (6 x His tag at N terminal). Recombinant proteins were produced in *E.coli* and total protein extracts were loaded on two identical gels. Both anti-AtMYB12 and anti-6 x His antibodies were used for the western blots.

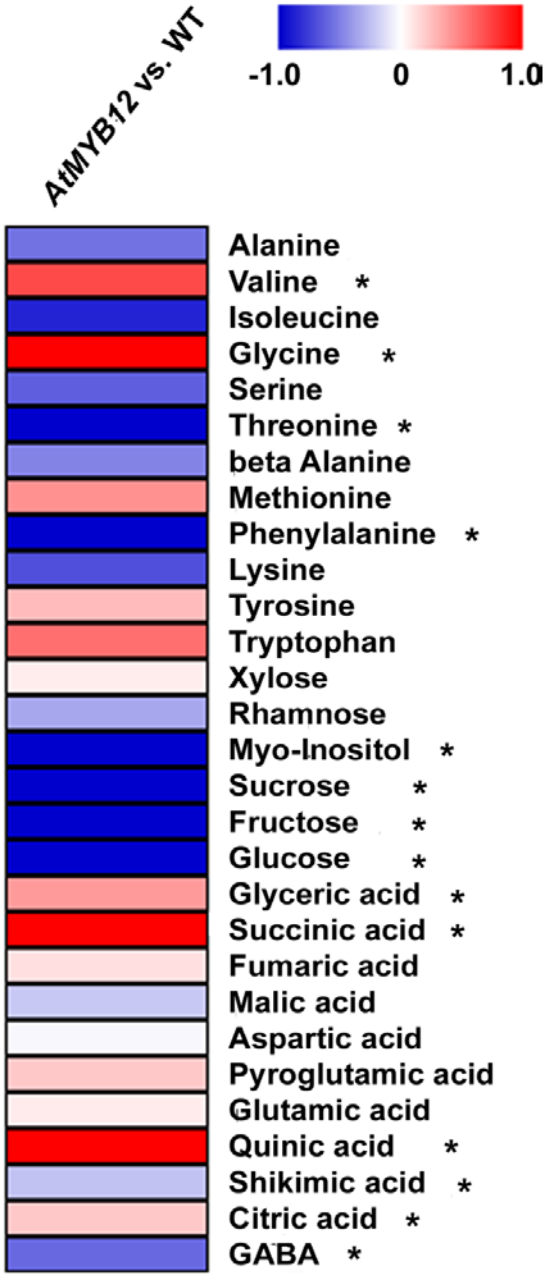

Supplementary Figure 2. Quantitative determination (nM g fresh wt^{-1}) of metabolite concentrations in fruit (10 dpb) of *AtMYB12* and *WT* tomato lines. The heat map compares the levels in *AtMYB12* tomato with those in WT fruit. Absolute values were scaled by log2. Asterisks indicate values that were determined by the Student's t test to be significantly different (p<0.05) (n=4, Student's *t* test).

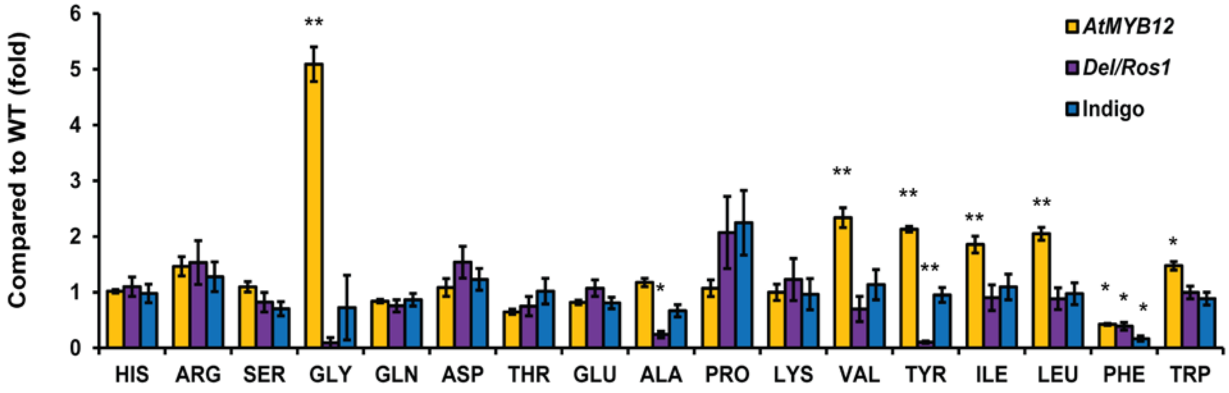

Supplementary Figure 3. ***AtMYB12*** **and** ***Del/Ros1*** **tomatoes showed different patterns of amino acid accumulation.** Fruit were harvested and analysed 7 days after breaker. Data show levels in different lines compared to those in WT tomatoes. Error bars indicate SEM (n=4). * (p<0.05) and ** (p<0.01) (Student's *t* test) indicate significant differences compared to WT fruit.

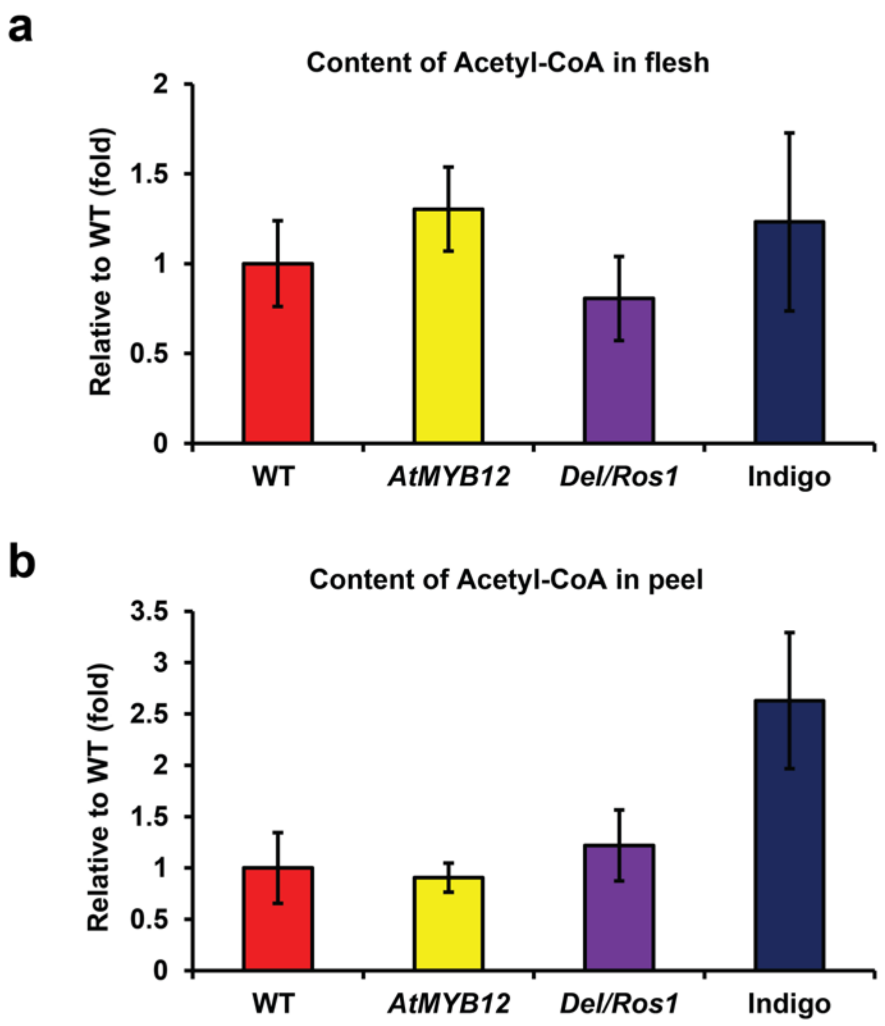

Supplementary Figure 4. Acetyl-CoA contents of different fruit. Fruit were harvest at 7 days after breaker. Both peel and flesh were analyzed for different lines. Data show the relative values compared to WT fruit for the same tissue. Error bars show SEM (n=4). No significant differences ($p<0.05$, Student's *t* test) were observed for any of the genotypes compared to WT fruit.

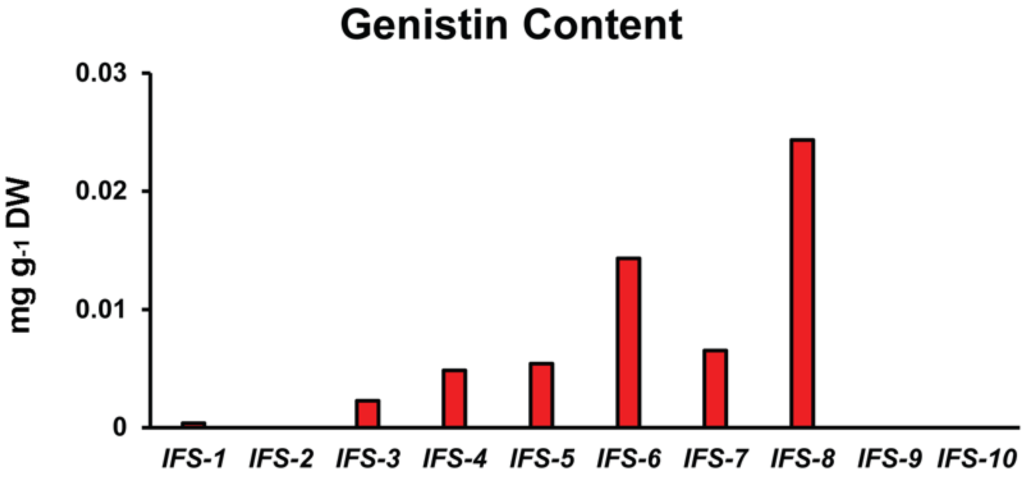

Supplementary Figure 5. Genistin content in primary *LjIFS* transformants of MicroTom tomato. Extracts of ten different T0 *IFS* tomato lines were analysed by LC-MS. For every plant, ripe fruit were pooled and analysed. Genistin, the major isoflavone detected, was used as a standard as a standard to evaluate isoflavone contents in different lines. Line 8 had the highest content of genistin and was selected for later experiments.

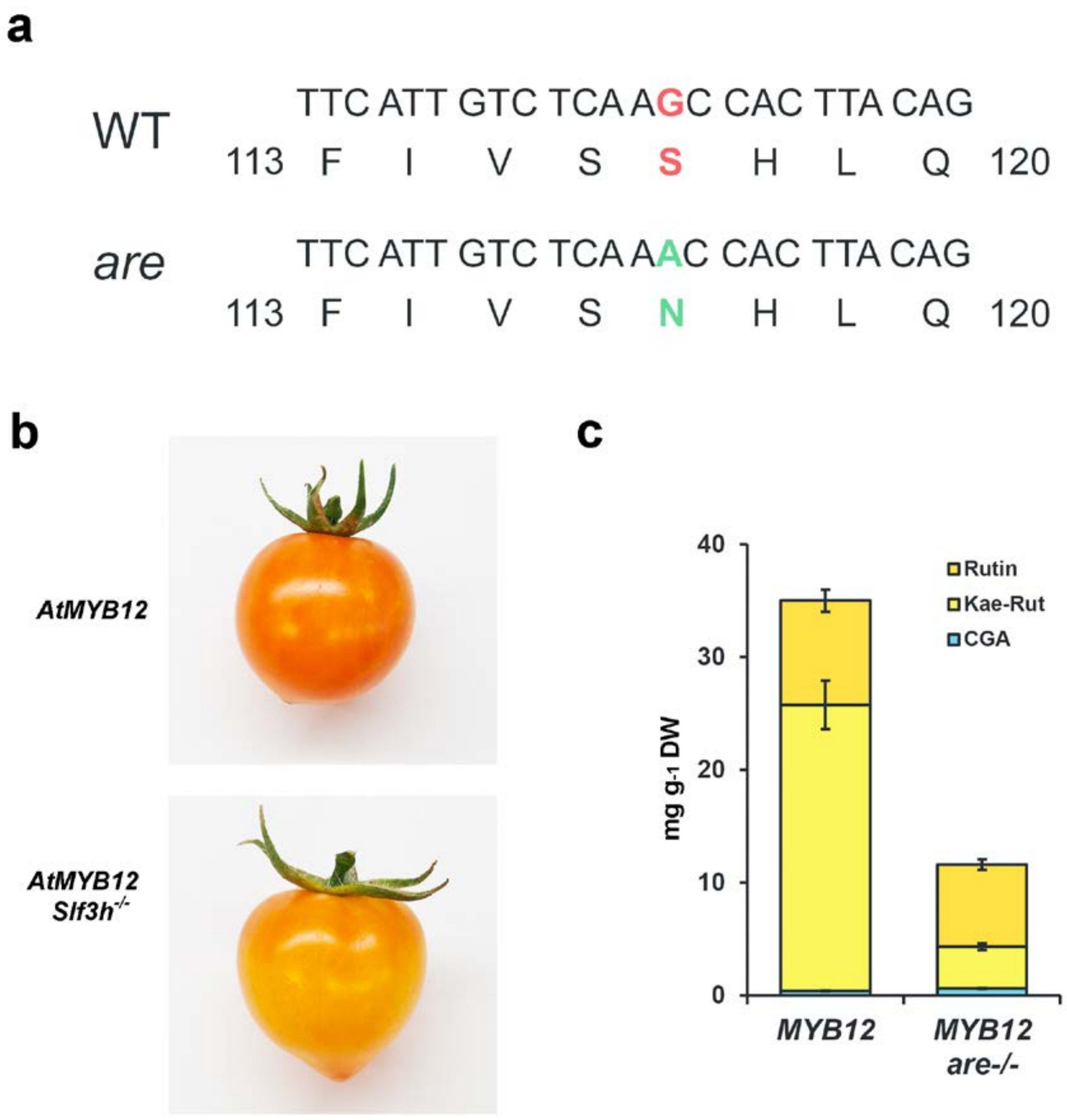

Supplementary Figure 6. The *are* mutation affects F3H activity. (**a**) The *are* mutant (3-073) has a G to A change in the coding sequence of the *SlF3H* (Solyc02g083860) gene. This causes a change in the predicted amino acid sequence of F3H from S to N. (**b**) Phenotype of *AtMYB12* and *AtMYB12 are*$^{-/-}$ tomatoes. The *AtMYB12* tomato was crossed with the *are* mutant. F2 plants were genotyped and plants with *AtMYB12* and *Are* ($^{+/+}$ or $^{+/-}$) or *AtMYB12 are*$^{-/-}$ were selected. Pictures were taken at 10 days after breaker. (**c**) The content of major flavonols in *AtMYB12* and *AtMYB12 are*$^{-/-}$ tomatoes. Fruit were harvested 10 days after breaker. Error bars indicate STDEV (n=3).

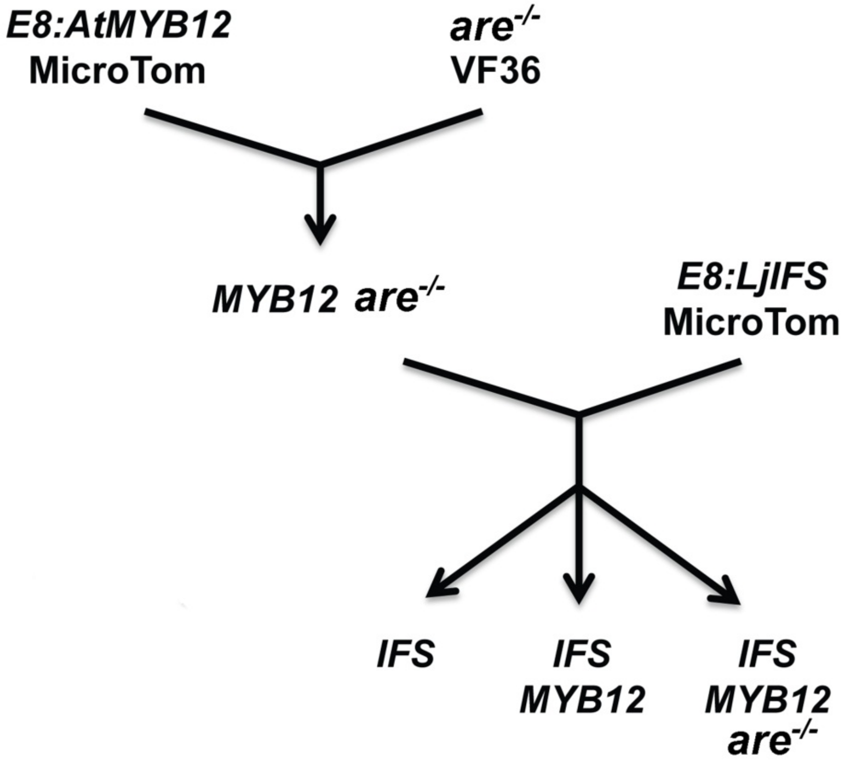

Supplementary Figure 7. Schematic representation of the crossing strategy for tomatoes with high isoflavone content. The *are*$^{-/-}$ mutant was first crossed with *AtMYB12* to make *AtMYB12 /are*$^{-/-}$ which was then crossed with the best *IFS* line. In the F_2 generation, segregation of different genes was analysed by genotyping.

Supplementary Tables

Supplementary Table 1. The ratio of $^{14}CO_2$ evolution from C1 and C6 of glucose by fruits of wild type and *AtMYB12* tomatoes. Ratios were calculated from the cumulative release of $C0_2$ after 1, 2, 3 , 4, 5 and 6 h incubation presented in **Fig 4a**. Each value represents the mean ± SEM of four independent samples; and asterisks indicate values that were significantly different at (p≤0.05, Student's *t* test) from WT

	1 hr.	2hr.	3 hr.	4 hr.	5 hr.	6 hr.
Wild Type	0.60 ± 0.06	0.42 ± 0.06	0.38 ± 0.04	0.39 ± 0.04	0.41 ± 0.04	0.45 ± 0.06
MYB12	0.53 ± 0.06	0.49 ± 0.05	0.51 ± 0.06	0.60 ± 0.10	0.68 ± 0.11	0.83* ± 0.13

Supplementary Table 2. Metabolism of [U-^{14}C] Glucose in pericarp disks from *AtMYB12* and WT fruit at 10dpb. After 4 h of incubation, disks were extracted and fractioned. Values are means ±SEM of determinations on four independent samples. Asterisks indicate values that were determined by the t-test to be significantly different ($P < 0.05$, Student's *t* test) from the wild type. FW, fresh weight

Parmeter	Wild Type			MYB12		
	Bq (gFW)$^{-1}$					
Total uptake	562.96	±	34.09	576.86	±	32.55
Insoluble						
Starch	4.74	±	0.56	3.94	±	0.36
Cell wall	0.60	±	0.07	0.64	±	0.13
Protein	0.19	±	0.03	0.16	±	0.03
Soluble						
Organic acids	118.90	±	18.89	127.48	±	17.57
Phosphates esters	105.98	±	12.32	121.39	±	6.44
Amino acids	94.37	±	15.72	129.81	±	13.67
Sucrose	238.18	±	27.77	193.44	±	26.49
	Bq (nmol)$^{-1}$					
Specific activity of hexose phosphates	0.21	±	0.01	0.26*	±	0.04
Metabolic flux	nmol (gFW)$^{-1}$ h^{-1}					
Starch	55.35	±	6.34	51.04	±	9.11
Cell wall	6.95	±	0.73	8.00	±	1.52
Sucrose	2781.28	±	318.90	2258.84	±	371.90
Carbohydrate oxidation	2491.10	±	112.85	2999.54*	±	135.00

Supplementary Table 3. Levels of major phenylpropanoids in WT, *AtMYB12*, *Del/Ros1* and Indigo (*AtMYB12* x *Del/Ros1*) tomatoes. Values are means ±SEM of determinations on three independent samples. * ($p<0.05$) and ** ($p<0.01$) (Student's *t* test) indicate significant difference compared to WT fruit.

Compounds (mg g^{-1} DW)	WT	*AtMYB12*	*Del/Ros1*	Indigo
Chlorogenic Acid	0.150 ± 0.002	1.035**±0.163	0.363 ± 0.154	2.118**±0.406
Kaempferol-3-Rutinoside	0.073 ± 0.002	4.050**± 0.469	0.276 ± 0.076	6.664*± 2.110
Rutin	0.894 ± 0.015	27.140**±2.070	5.183 ± 1.644	16.585**±0.981
Quercetin-(Coumaroyl)-Rutinoside	NA	NA	2.97**± 0.62	30.21**± 2.06
Myricetin-(Coumaroyl)-Rutinoside	NA	0.68**± 0.03	1.19**± 0.19	25.43**± 3.41
Methylmyricetin-(Coumaroyl)-Rutinoside	NA	NA	0.72**± 0.12	16.43*± 4.55
Delphinidin-(Coumaroyl)-Rutinoside-Glucoside	NA	NA	1.294**± 0.187	1.154**± 0.011
Petunidin-(Coumaroyl)-Rutinoside-Glucoside	NA	NA	1.477**± 0.163	2.857**± 0.218
Petunidin-(Feruloyl)-Rutinoside-Glucoside	NA	NA	0.420 ± 0.211	0.922**± 0.102
Malvidin-(Coumaroyl)-Rutinoside-Glucoside	NA	NA	NA	0.598**± 0.011

Supplementary Table 4. Contents of major phenylpropanoids in WT, *StSy*, *AtMYB12* and *AtMYB12 StSy* tomatoes. Values are means ±SEM of determinations on three independent samples. * (p<0.05) and ** (p<0.01) (Student's *t* test) indicate significant differences compared to WT fruit.

Compounds (mg g^{-1} DW)	WT	*StSy*	*AtMYB12*	*AtMYB12 StSy*	*AtMYB12 StSy are-/-*
Chlorogenic Acid	0.117 ± 0.008	0.192 ± 0.024	0.912**± 0.022	0.180 ± 0.106	0.200 ± 0.135
Kaempferol-3-Rutinoside	0.351 ± 0.020	0.118* ± 0.014	20.934**±0.450	0.779* ± 0.089	1.393 ± 0.267
Rutin	1.649 ± 0.153	0.118**±0.009	12.770**±0.382	0.129**± 0.016	1.011 ± 0.213
Resveratrol	0.018 ± 0.007	0.519**±0.044	0.011 ± 0.004	0.447 ± 0.106	0.297 ± 0.130
Piceid	0.011 ± 0.011	0.688**±0.056	NA	2.824**± 0.074	2.597*± 0.418
Resveratrol-Glucoside Isoform	0.047 ± 0.003	0.608**±0.051	0.038 ± 0.038	1.513**± 0.081	1.717*± 0.244
Resveratrol-di-Glucosides	0.053 ± 0.053	0.379* ± 0.070	NA	0.487**± 0.069	1.004**± 0.124
Methyl-Resveratrol-Glucosides	0.005 ± 0.005	0.036**±0.003	NA	0.992**± 0.070	0.306 ± 0.088

ǂ this could be an under estimate due to difficulties of extracting resveratrol and resveratrol derivatives completely by the protocol used.

Supplementary Table 5. Content of major phenylpropanoids in WT, *IFS*, *AtMYB12 IFS* and *AtMYB12 IFS are*$^{-/-}$ tomatoes. Values are means ±SEM of determinations on three independent samples. * ($p<0.05$) and ** ($p<0.01$) (Student's *t* test) indicate significant differences compared to WT fruit.

Compounds (mg g^{-1} DW)	WT	*IFS*	*AtMYB12 IFS*	*AtMYB12 IFS are-/-*	*IFS are-/-*
Chlorogenic Acid	0.102 ± 0.006	0.280* ± 0.029	0.589** ± 0.040	0.495** ± 0.025	0.150* ± 0.017
Kaempferol-3-Rutinoside	0.298 ± 0.027	0.105* ± 0.008	41.693**±2.774	3.88** ± 0.283	0.094* ± 0.010
Rutin	1.458 ± 0.093	0.799**± 0.096	22.197**±1.258	3.032** ± 0.200	0.148**±0.017
Genistein	0.105 ± 0.007	0.278* ± 0.032	0.220** ± 0.013	0.738** ± 0.053	0.160* ± 0.018
Genistin	0.011 ± 0.001	NA	10.854**±0.706	76.221**±4.078	0.170**±0.012
Genistein-di-Glucosides	NA	0.013 ± 0.007	NA	1.729* ± 0.127	NA

ǂThese data were derived from the best lines segregating in the F2 population.

Supplementary Table 6. Oligonucleotides used in ChIP-qPCR experiments

ID	Symbol	Full Name	ChIP-qPCR Primer Sequence (5'-3')
Solyc02g093830	*G6PD*	Glucose-6-phosphate 1-dehydrogenase	GTCAGTGGCTCTTATGATTCTGG<br>GTCTTCTGGATGATCCTTCTGTG
Solyc05g012110	*PGLS*	6-phosphogluconolactonase	GTGGTATCCCACAAGGTAAAGTT<br>AATACAACATGAATTTTGCTATGCT
Solyc04g005160	*6PGD1*	6-phosphogluconate dehydrogenase	ATCCAGTACAAATGGAAGAATGG<br>TTCCCTTACAATGAAATGAAAACA
Solyc05g010260	*6PGD2*	6-phosphogluconate dehydrogenase	TCGTGACTTTTGGTTCTTTCTTTAG<br>ATGATATTTCTGCTAGCATCCACTC
Solyc05g008370	*Rpi*	Ribose-5-phosphate isomerase	ATTCCACAAGTTCCAATCTTGATAA<br>GATGAAAAATCAAGAAACAGAGGAA
Solyc05g007260	*Rpe*	Ribulose-phosphate 3-epimerase	TGGAGCATAATTAAATCAAACCCTA<br>GCATTCAATAATTAAAACTAGGGAAAA
Solyc10g018300	*TKT*	Transketolase 1	AGGAGCCAAAATAAGCAAAATAAGT<br>ATGTTTTGTGGATGAAATTGAAAAT
Solyc00g006800	*TALDO1*	Transaldolase	AAACCAAAAGAGTAATTAGAAATACCA<br>TTTAAGGGTTGTTGTACCTCACTTAT
Solyc07g042550	*SUS1*	Sucrose synthase	TAAAAGAGCTTAGCAGGCAAACA<br>GGAATGGTAGAGGTCCCTTTTTA
Solyc12g095760	*PFK1*	Diphosphate-fructose-6-phosphate 1-phosphotransferase	CAAAACAATAAGGACCATTTCCATC<br>CCCGCTCTGAAAATACGGTTTAT
Solyc03g111010	*GADPH*	Glyceraldehyde-3-phosphate dehydrogenase	ACGATAGTGAAAATCCTCAACGA<br>AACAGAGTAGTGGAGTTGTTGGTG
Solyc04g009030	*GADPHL*	Glyceraldehyde-3-phosphate dehydrogenase like	TCATTTTGTAAGTTGAAGTGTTCG<br>CGCTCAACTTGTCTTTAAATGCT
Solyc04g072800	*PGM*	2 3-bisphosphoglycerate-dependent phosphoglycerate mutase	GGTAACTCTGGATTGTGTCCTGA<br>CAAAAACTTCCTCCTCTCAACAA
Solyc03g114500	*ENO*	Enolase	ATTTTCGTACGCTCGTGTCC<br>TGCTTTGGAGGAGACGTTAG
Solyc04g074480	*DAHPS*	3-deoxy-7-phosphoheptulonate synthase	CACATATTTTTCCTAAAATACCCTTT<br>GGAATGGGGTTTTATAAGGAGGT
Solyc02g083590	*DHQS*	Dehydroquinate synthase	GATGGATCACTCGTCCAGTAAAG<br>GTCAGGTTGATCAAGAAGTCCAG
Solyc06g084460	*DHQD*	Dehydroquinate dehydratase	ACTTGATTTGGCAAAAAGAAAAGAT<br>AATCCTACTTAGAGACATTGTTTTTGT
Solyc01g067750	*SHD*	Shikimate dehydrogenase	CACGTTTGGTACTCTTGAAGTGG<br>GTATCTGGTCCCAACTGTCTGAA
Solyc04g051860	*SK*	Shikimate kinase	GGACGATGTGTATACCTTGTTGG<br>CTCTATCAGCCTGTCACAGTCAA
Solyc05g050980	*EPSPS1*	3-phosphoshikimate 1-carboxyvinyltransferase	CCTCCTATGTGGTTTGCGAGC<br>AACGAAAATTTGCGGTTGCT
Solyc01g091190	*EPSPS2*	3-phosphoshikimate 1-carboxyvinyltransferase	CCAAACAGTTAAATAAGTCAAAAATCA<br>TTATTAGAGCCTGTTTGGCTCAG
Solyc04g049350	*CS*	Chorismate synthase	TAGAAATCTGCCCCTCTTGAAAT

			TAGACGAGTCATTTGATCCACCA
Solyc02g088460	*CM1*	Chorismate mutase 1	GGTAGTAGGAGGATATCGCATCA<br>TGGGTTTTGGAGTTAGGTGAGT

ID	Symbol	Full Name	ChIP-qPCR Primer Sequence (5'-3')
Solyc04g054710	*PAT*	Prephenate aminotransferase	TACTTCACCAACCAAAGAGGAAA<br>GTGAGTGAAACAGAAGAGGGTTG
Solyc07g007590	*ADT*	Prephenate dehydrogenase family protein	GAAATCGAAGCCGATTTACACAA<br>AGAGGACTTTTTCCACGAAGCAC
Solyc09g007920	*PAL5A*	Phenylalanine ammonia-lyase	TGGCTATAATTAATCTTCCAACAACCA<br>AGAGAGGAACAAAGGATGGTAGG
Solyc09g007910	*PAL5B*	Phenylalanine ammonia-lyase	TGGCCATGATTAATCTTTCAACAA<br>GGGGGAACTAATAGATGGTAGGTAAA
Solyc09g007900	*PAL5C*	Phenylalanine ammonia-lyase	TTGAAGTCAAAGGACACAATAATGGA<br>ATATGTGAGAGGGGGTTGCTAGG
Solyc09g007890	*PAL5D*	Phenylalanine ammonia-lyase	ACCAACTCACCCCTCACATATC<br>ATTTTTGTTGTTGTTTTGGTGGTG
Solyc10g086180	*PAL*	Phenylalanine ammonia-lyase	CGGTCCATATAATTCCTTATGTCCA<br>AGGTGAGTTGATAAGGGGTCCAT
Solyc03g117870	*4CL*	4-coumarate CoA ligase	TTGTATCCCCCAAAAAGGAAAAA<br>CCTGTGGTAGGTGAAAGGGAGTT
Solyc06g035960	*4CL-Like*	4-coumarate-CoA ligase-like	ATTGAGGAAATATGCCCTCTCCA<br>GCGGCAATATAAATGCAATGAAG
Solyc09g091510	*CHS-1*	Chalcone synthase -1	TCTTAAATTTAGAAAGGAAAACCAAAA<br>TCCAAAAGAAAAAGAATGGTAGC
Solyc05g053550	*CHS-2*	Chalcone synthase -2	ACGAAAAAATAAGAGTTGGGGAAA<br>CATAACAAACTGGTAGGCGGTAG
Solyc05g010320	*CHI*	Chalcone-flavonone isomerase	TCTCGAAATACGAAATAATTGAGG<br>GTACGGTTAATGGCTCACAGTTC
Solyc05g052240	*CHIL*	Chalcone-flavonone isomerase like	CTTTCCAAATACTCGTGTCAAGC<br>AGCTACCACACAATCTACCTTCC
Solyc02g083860	*F3H*	Flavanone 3-hydroxylase	CATAATTTTCAAACCAAGTATGGTGGT<br>CGTGCCAACTAACAACATTTCAC
Solyc11g013110	*FLS*	Flavonol synthase	AATTAAAGGCAAAAAGTAGGTTTGAA<br>TTATAGGAAAGTGACGCGGTGT
Solyc02g089770	*DFRL1*	Dihydroflavonol-4-reductase like	AACTCCTGCAATTAGTCTTTCACA<br>CATCCGAGCATATAAGAAACCAA
Solyc01g068080	*DFRL2*	Dihydroflavonol-4-reductase like	AGTACAACGCGGCGTATCACTAT<br>TTCTATGGCTTCGAGTTAGAAAAA
Solyc08g080040	*ANS*	Anthocyanidin synthase	ATGCATGGTGGTTGTTGAATGTT<br>AATTCTTCCCCTTTAGGGTGATTT
Solyc10g083440	*3GT*	UDP flavonoid 3-O-glucosyltransferase	ACAAAATCCTCATGCTATCAACC<br>CTTTCTTTGTTGGTGGTTAAAAA
Solyc03g117600	*HCT*	Hydroxycinnamoyl-CoA shikimate/quinate hydroxycinnamoyltransferase	TTTTCTTAAATTACGTATCAGGTTTTT<br>TGAGTTTCTTATATAGTATTGGGAAGG
Solyc10g078240	*C3H*	*p*-coumarate 3' -hydrolase	ACCAGCCACGTGATTGAGAAAT<br>TTGCGTTTGGAGTTTTGGTAGGT

Supplementary Table 7. Oligonucleotides used in RT-qPCR experiments

ID	Symbol	RT-qPCR Primer Sequence (5'-3')
Solyc02g093830	*G6PD*	GTCAGTGGCTCTTATGATTCTGG<br>GTCTTCTGGATGATCCTTCTGTG
Solyc05g008370	*Rpi*	TCTTCCTCTGTTTCCCCTGTTA<br>CTGTTGACCCTGTACCTAAACCA
Solyc10g018300	*TKT*	GTCTAGGAGAAGACGGTCCAACT<br>TAAGCTCCTGCTGTCTCATTACC
Solyc07g042550	*SUS1*	GTTAGGTGTAACACAGTGCACCA<br>AGCTGTGAACTGAGCTGAGAAGT
Solyc12g095760	*PFK1*	GCACTCTGCAGTCACATCCTAAT<br>TCAGCCCTAGCTTGAACTGTATC
Solyc04g072800	*PGM*	GACAAGCTAACATCCCAAGAGGT<br>AGGACTTCCTCTGCGAATATACC
Solyc03g114500	*ENO*	GGATGTAGCAGCATCTGAGTTCT<br>GGCTCTGAGCACTAAGTACATGA
Solyc04g074480	*DAHPS*	CCTTCACTATGATTGCTCTGCTC<br>TCACTCACCTTAATACCGAGAGG
Solyc02g083590	*DHQS*	GATGGATCACTCGTCCAGTAAAG<br>GTCAGGTTGATCAAGAAGTCCAG
Solyc01g067750	*SHD*	CACGTTTGGTACTCTTGAAGTGG<br>GTATCTGGTCCCAACTGTCTGAA
Solyc04g051860	*SK*	GGACGATGTGTATACCTTGTTGG<br>CTCTATCAGCCTGTCACAGTCAA
Solyc04g049350	*CS*	CAGGGCGGTATATCAAATGG<br>GCGATGAGTTCTGTTTCGTG
Solyc02g088460	*CM1*	CAGTATCCAAAGGTTCTGCACTC<br>TGCCTTCCTTCACTAATCTTGG
Solyc07g007590	*ADT*	GTCACAAGTCAGTCACCACCAT<br>AGCCTACCTGACTGAGCATATTG
Solyc00g282510	*PAL*	AATTGCTTCGAGTCGTGGATAG<br>ACAAGGACTTGTCTCAGCTTCTG
Solyc09g091510	*CHS-1*	CCTTTATTTGAACTCGTCTCAGC<br>CAGGAACATCCTTGAGTAAGTGG
Solyc05g053550	*CHS-2*	CGGGCTACTAGGCAAGTTTTAAG<br>CCTGTGGTACTAAGCCCTTCTTT
Solyc05g010320	*CHI*	TTGTCAACTCGGTCTAATGTGTC<br>TAAAGTGGGACCTTATTGCACAC
Solyc02g083860	*F3H*	ATGGATGAGCCGATTACATTTG<br>TGGCCTCTTCAGTTTGTATCTTC
Solyc11g066580	*F3'5'H*	CTCAACGCCACTAAATCTCCCTA<br>TTGCCCATATGTTGACACTAAGC
Solyc03g115220	*F3'H*	TATAGCTGGGACAGACACATCCT<br>CTACTTTGTCGATCTCCTGTTGG
Solyc02g085020	*DFR*	GACTTGCCGACAGAAGCAAT<br>GTGCATTCTCCTTGCCACTT
Solyc08g080040	*ANS*	ACGAACAGGATTTTGCTGCT<br>TTTGAGCTCAGCAACTGCAT
Solyc11g013110	*FLS*	TGTCCCATATCACCCTTCTTGTC<br>TCACCAATGTGGACAATTATAGCA
